# Supplementary material for: Association between oral microbiome and seven types of cancers in East Asian population: a two-sample Mendelian randomization analysis
Source: Front Mol Biosci. 2023 Nov 21;10:1327893. doi: 10.3389/fmolb.2023.1327893 (PMC10702768; doi:10.3389/fmolb.2023.1327893)
Supplement: Supplementary file 1 [file DataSheet1.ZIP › Supplementary Materials/MR plots for tongue/tongue═╝/Pancreatic cancer/╥╚╧┘░⌐_╔╕╤í╜ß╣√/pheno.46_to_pancreatic cancer_forest.pdf]

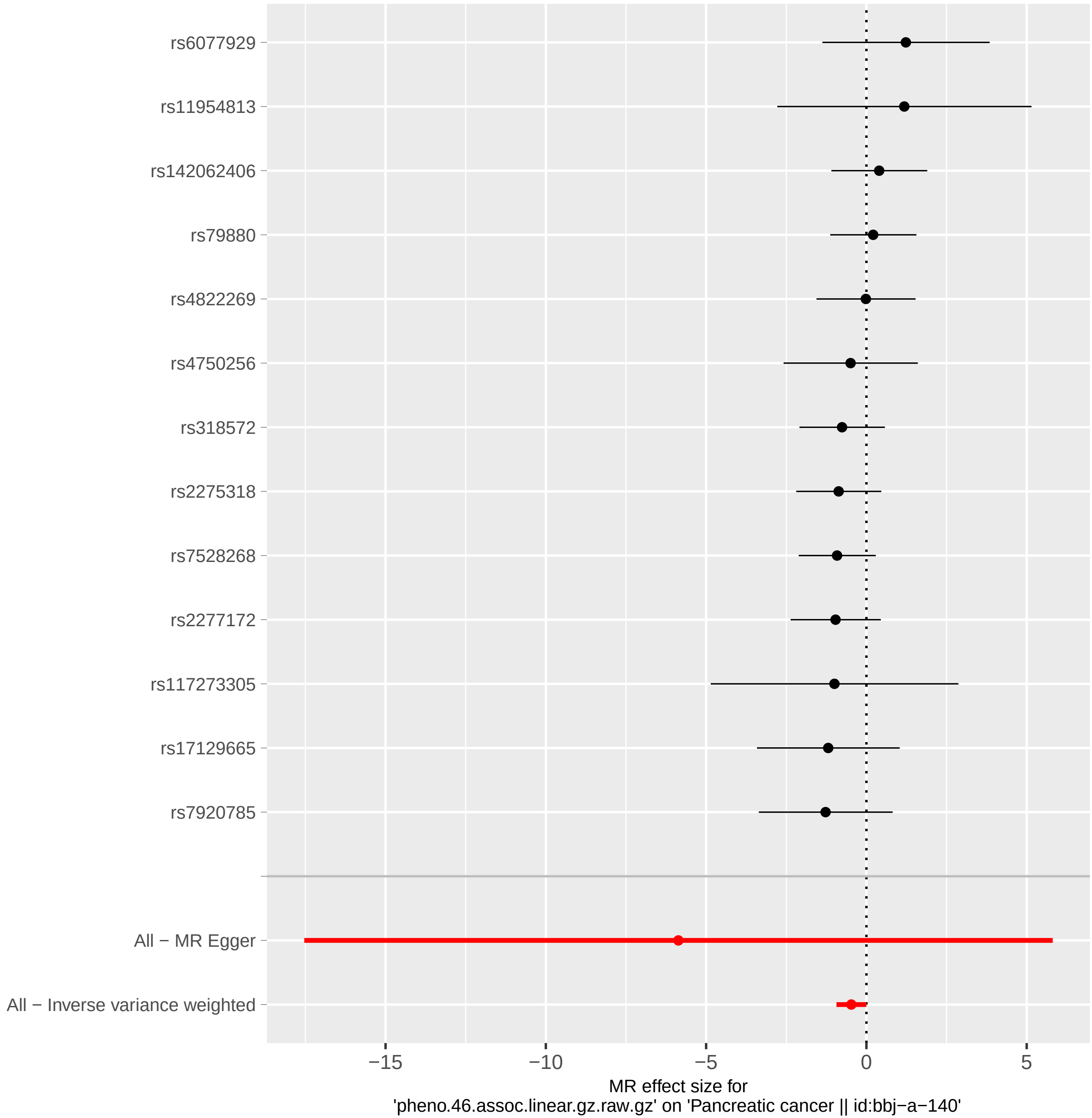

rs6077929
rs11954813
rs142062406
rs79880
rs4822269
rs4750256
rs318572
rs2275318
rs7528268
rs2277172
rs117273305
rs17129665
rs7920785
All − MR Egger
All − Inverse variance weighted
−15
−10
−5
0
5
MR effect size for
'pheno.46.assoc.linear.gz.raw.gz' on 'Pancreatic cancer || id:bbj−a−140'
